# Advanced glaucoma secondary to bilateral idiopathic dilated episcleral veins – a case report

**DOI:** 10.1186/s12886-018-0892-1

**Published:** 2018-08-23

**Authors:** Xin Rong, Mei Li

**Affiliations:** Department of Ophthalmology, Peking University First Hospital, 8 Xi Shi Ku Street, Xi Cheng District, Beijing, 100034 People’s Republic of China

**Keywords:** Episcleral vessel dilation, Secondary glaucoma, Trabeculectomy

## Abstract

**Background:**

Idiopathic elevated episcleral venous pressure (IEEVP) or idiopathic dilated episcleral veins (IDEV) is a rare abnormality, and thus therapeutic treatment for this condition rarely is discussed. We report a case of a patient with bilateral glaucoma secondary to IDEV for whom intraocular pressures (IOPs) were controlled successfully by trabeculectomy.

**Case presentation:**

A 50 year-old female with a complaint of persistent red eyes for over 30 years, presented with numerous tortuous and engorged episcleral vessels in both eyes (OU), open angles OU with spontaneous blood in Schlemm’s canal 360 degrees bilaterally. Orbital color Doppler examination showed the superior ophthalmic veins to be of normal calibre, with no reversal of flow. Head MRI with contrast and cerebral angiography were negative for arteriovenous fistula. Coronary angiography, color Doppler echocardiography and chest radiographs were within normal limits. A diagnosis of secondary glaucoma and IDEV was made. Neither anti-glaucoma medications, nor laser trabeculoplasty reduced the patent’s IOP effectively. Only after trabeculectomy was performed in each eye, were IOPs successfully controlled.

**Conclusions:**

This case serves to remind clinicians of the importance of identifying and evaluating thoroughly patients with episcleral vessel dilation in non-inflamed eyes with no known cause. A misdiagnosis or missed diagnosis of long-term elevated intraocular pressure may result in significant damage to the optic nerve. In addition, when performing filtration surgery it is crucial that the ophthalmologist control the IOP and make an effort to prevent choroidal effusion.

## Background

Resistance to aqueous humor outflow arises not only from tissues within the sclera, including the trabecular meshwork and Schlemm’s canal, but also from certain extraocular tissues, such as episcleral veins. Elevation of episcleral venous pressure may impede aqueous humor outflow, increase intraocular pressure (IOP) and lead to secondary glaucoma. The clinical findings of elevated episcleral venous pressure include dilated episcleral veins without inflammation, an open angle with blood in Schlemm’s canal, and an associated elevated IOP, which may lead to characteristic optic nerve damage and visual field loss. Patients with obstructive pathology in the aqueous circulating systems outside the eyeball may present with elevated episcleral venous pressure, and when a patient with elevated episcleral venous pressure presents without any underlying cause, this is referred to as idiopathic elevated episcleral venous pressure (IEEVP) [1]. It is also termed idiopathic dilated episcleral veins (IDEV) as there is no clinically available method of measuring the episcleral pressure [1–3]. IDEV is not a common disease and thus therapeutic treatment rarely is discussed. This manuscript describes one case of a patient with bilateral glaucoma secondary to IDEV for whom IOPs were controlled successfully by trabeculectomy.

## Case presentation

A 50 year-old Chinese female with a complaint of bilateral blurred vision of 2 years duration was referred to us after elevated IOP of 1 week duration was documented. History revealed no family history of glaucoma, no trauma to the head or neck, and no headache. The patient did not have diplopia, pulsatile tinnitus, nor pulsation of the orbit. She reported having persistent red eyes for over 30 years, for which she intermittently used several anti-inflammatory eye drops in both eyes (OU), all of which were ineffective.

**Fig. 1:**
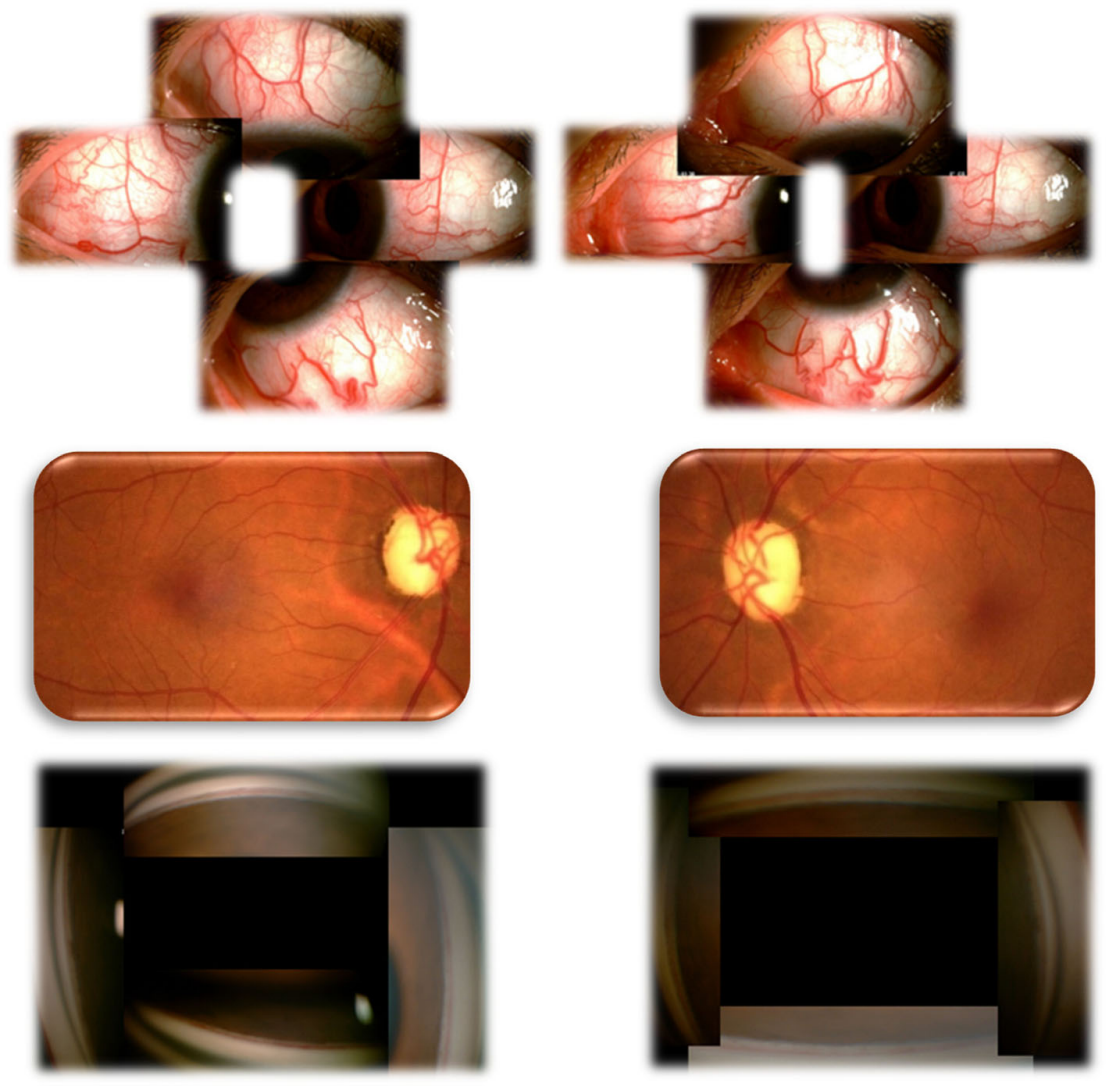
External examination, fundi appearances and gonioscopic characteristics of both eyes on the day of initial presentation, left side is right eye, right side is left eye. The eyes showed numerous tortuous and engorged episcleral vessels, a vertically elongated cup, white optic nerve heads, normal retinal vessels, and open angles with spontaneous blood in Schlemm’s canal 360 degrees bilaterally

**Fig. 2:**
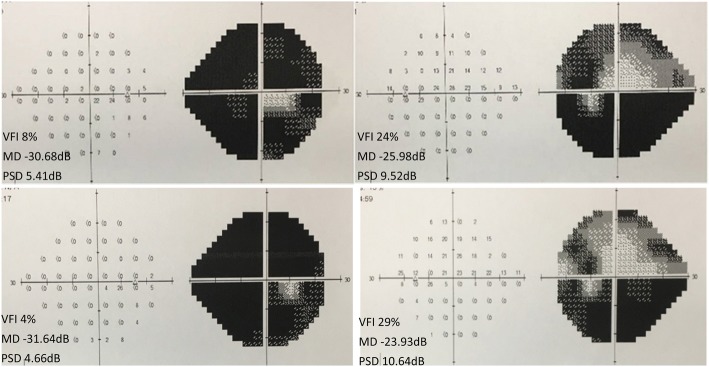
The first row shows the visual field of each eye on the day of the initial visit. Tubular vision is indicated in both eyes, but is more prominent in right eye. One year later, as shown in the second row, the visual field of the right eye is constricted further

On examination, uncorrected visual acuity (UCVA) was 20/40 right eye (RE) and left eye (LE), best corrected visual acuity (BCVA) was 20/20 (− 1.00D OU), IOPs were 36 mmHg RE and 30 mmHg LE. Findings of the adnexa were unremarkable, ocular motility was normal and no relative afferent pupillary defect was detected. No carotid or ocular bruits were heard. Anterior segment examination revealed numerous tortuous and engorged episcleral vessels bilaterally (Fig. 1), while the conjunctival vessels were normal with no chemosis. Anterior chambers were deep without any inflammatory reaction. Dilated fundus exam revealed optic nerve rim loss with a cup-to-disc ratio of 0.8 H × 1.0 V OU, without retinal vessel dilation or tortuosity (Fig. 1). Axial lengths measured 23.46 mm RE and 23.58 mm LE, and an ultrasound B-scan showed no thickening of the sclera in either eye. Tubular visual field was present bilaterally but more prominent RE (Fig. 2). Gonioscopic examination showed open angles with spontaneous blood in Schlemm’s canal 360 degrees OU (Fig. 1). Thus, secondary glaucoma, dilated episcleral veins and refractive error were the initial diagnoses. Various anti-glaucoma eye drops were administered, while scheduling of other relevant diagnostic procedures were arranged.

**Fig. 3:**
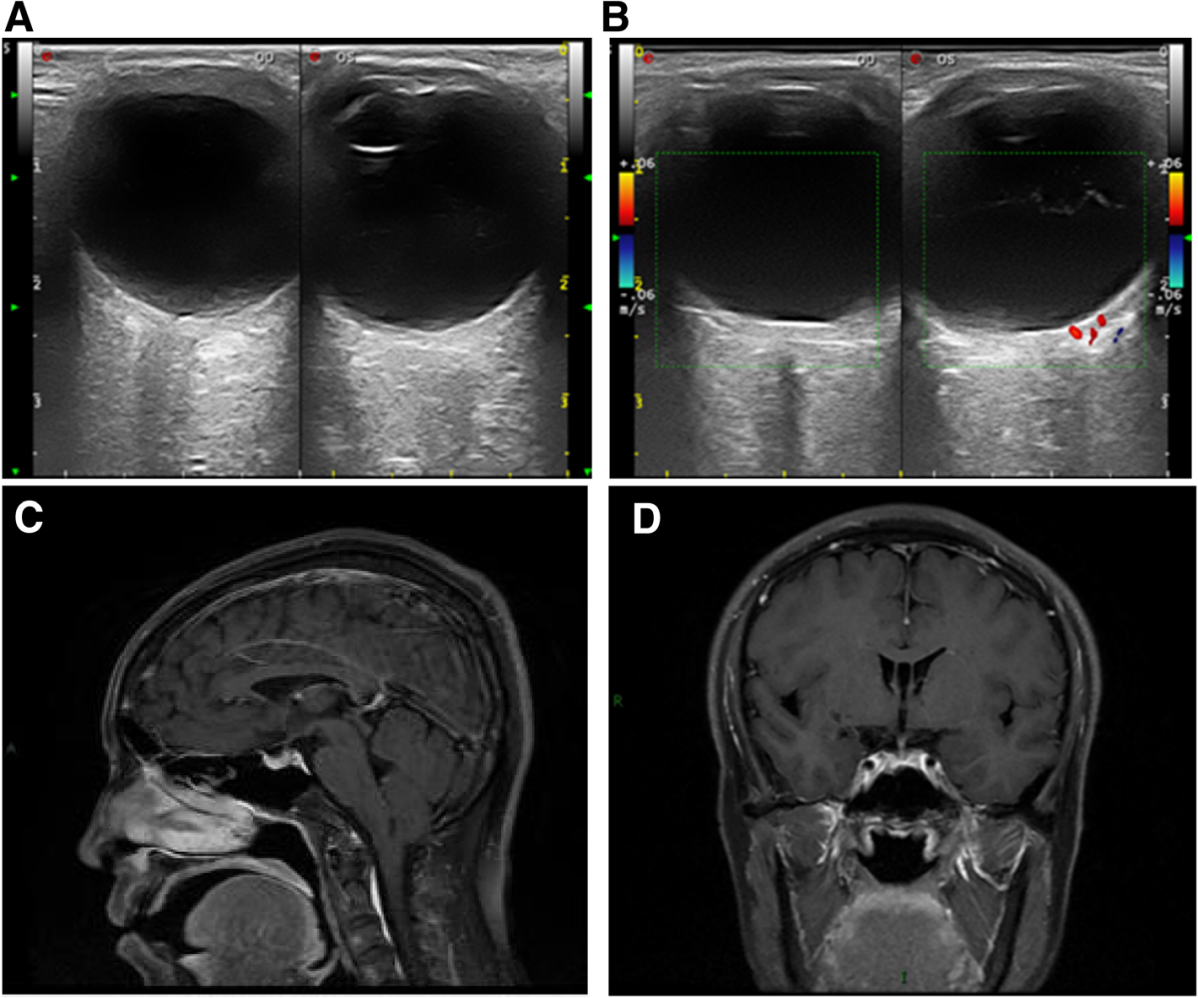
**a** B scan of two eyes revealed normal scleral thickness. No occupied lesions were indicated on sonogram. **b** Orbital color Doppler imaging showed no signs of abnormal blood flow, nor any reversal of flow. The head enhanced axial MRI image (**c**) and coronal scanning image (**d**), revealed no blood transfer between the cavernous sinus and intracranial artery in our case. No vascular malformation or space occupying lesion was observed

Orbital color Doppler examination showed that the superior ophthalmic veins were of normal calibre in each orbit, with no reversal of flow (Fig. 3). Head MRI with-and-without contrast was normal, without blood transfer between the cavernous sinus and intracranial artery, and without vascular malformation or space occupying lesions (Fig. 3). Cerebral angiography, which was the most valuable method of diagnosing head or orbit vasculopathy, was also negative for arteriovenous fistula. Coronary angiography, color Doppler echocardiography and chest radiographs were within normal limits. Due to an absence of thyroid history, proptosis, ptosis and facial cutaneous angiomatosis, as well as negative radiologic findings and systemic manifestations for superior vena cava syndrome, the clinical diagnosis of glaucoma secondary to IDEV was established in both eyes.

**Fig. 4:**
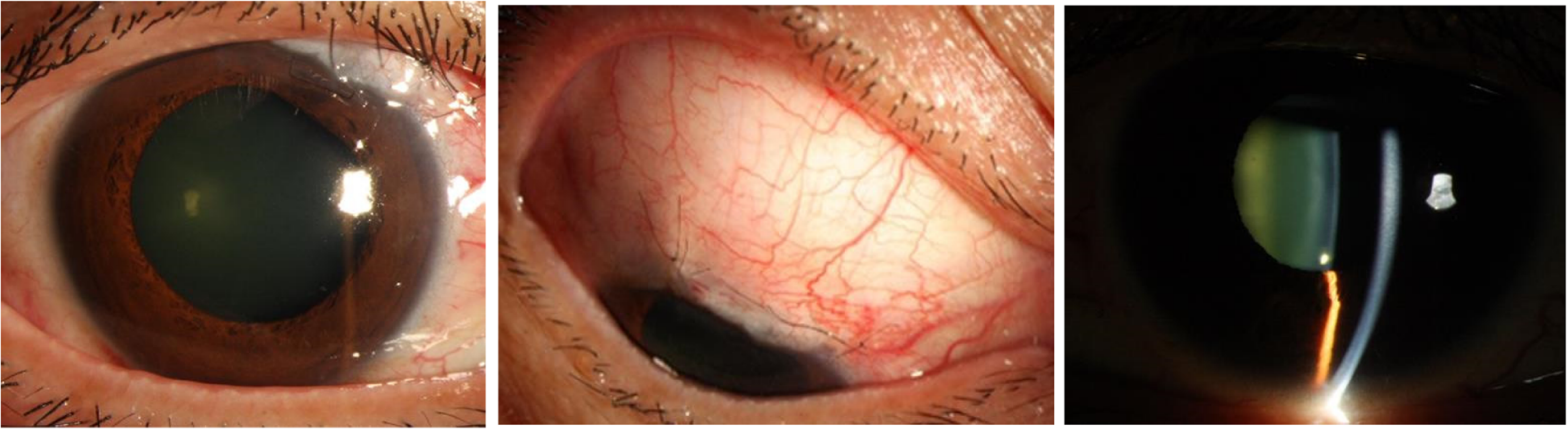
External examination of the right eye, photographed 5 days after trabeculectomy showing a diffuse but congestive bleb and a deep anterior chamber

Three days after the initial presentation (July 19, 2016), the intraocular pressure reduced to 24 mmHg RE and 21 mmHg LE with use of carteolol hydrochloride 2% and brinzolamide 1% twice a day and travoprost 0.004% at bedtime OU. Although surgery was recommended, the patient declined. After 10 days (July 29, 2016), the patient visited another hospital where micropulse diode laser trabeculoplasty (MLT) was performed on the right eye, resulting in minimal IOP decrease in the days following. When surgery was recommended again, the patient still refused. One year later (July 5, 2017), the patient was referred to us again for uncontrolled IOP 18–24 mmHg RE and 15-23 mmHg LE with medication prescribed previously, and a further decreased visual field RE (Fig. 2). With the patient’s consent for surgery finally obtained, trabeculectomy was performed (July 7, 2017) on her right eye. 5-Fluorouracil 5 mg was injected subconjunctivally at the site of incision (25 mg/ml in a volume of 0.2 ml) immediately before the surgery. During the procedure, adjustable scleral sutures were used to close the scleral flap more tightly than usual. After surgery, in addition to antibiotic and anti-inflammatory eye drops, other topical drugs were applied to the eye, including atropine 1% daily for 2 weeks, tropicamide 0.5% one administration (5 min*4) in the morning daily for 4 weeks and prednisone acetate 40 mg daily for 7 days. The IOP decreased to 12-18 mmHg with a relatively deep anterior chamber. At day 5-postop (Fig. 4), a diffuse but congestive bleb had formed. In response, 5-fluorouracil 5 mg was injected beneath the conjunctiva near the bleb twice a week for 3 weeks. The eyeball was massaged one to three times a day after day 7-postop. Two adjustable stitches on the scleral flap were removed on the sixth and ninth postoperative day respectively. Four months later, a similar surgical procedure was performed on the left eye. During the nine-month post-operative follow-up period for the right eye, and the five-month follow-up period for the left eye, the IOP of each eye ranged between 8 and 11 mmHg with a diffuse bleb. No apparent progression of field loss was noted, but persistent episcleral vessel dilation, as well as congestion in Schlemm’s canal, were observed.

## Discussion

IEEVP or IDEV is an uncommon cause of secondary open-angle glaucoma, first described by Minas and Podos [4], and has no apparent predilection in regard to age or gender. It may be unilateral or bilateral in presentation, and there may be asymmetric involvement. In patients with IEEVP, congenital vascular abnormality and familial predisposition have been hypothesized as the cause; however, some authors suggest that the red eye is acquired. The onset of vessel dilation varies from late teens to the third or fourth decade of life. The diagnosis of IEEVP or IDEV is one of exclusion, as it may be considered only when careful history, ophthalmologic examination and imaging findings fail to identify any plausible aetiology for the elevated episcleral venous pressure.

After the aqueous humor flows into the episcleral venous plexus, it flows first into the anterior ciliary vein, and then enters the superior ophthalmic vein. Then, above the annulus of Zinn, it enters the cavernous sinus from behind the superior orbital fissure, flowing into the superior vena cava through the internal jugular vein, and finally drains into the right atrium. Any obstruction of the drainage pathway may elevate episcleral venous pressure causing episcleral vessels dilation. Diseases in which such obstructions may be present include carotid-cavernous-sinus fistula, thyroid related ophthalmopathy, Sturge-Weber syndrome, cavernous sinus thrombosis, superior vena cava syndrome, scleritis, and head/orbital tumors [5]. All these conditions need to be excluded before a diagnosis of IEEVP or IDEV is established. Dural arteriovenous shunts or fistulas, a sub-type of arteriovenous fistulas, also termed indirect cavernous-sinus fistula, is the most common misdiagnosis for IEEVP or IDEV, as the flow through this fistula is so minute that it cannot be detected by neuro-radiologic examination. In diagnosing an arteriovenous fistula, cerebral angiography is the gold standard, and orbital color Doppler ultrasound is also helpful as a non-invasive method to confirm dilation of the superior ophthalmic vein or other veins when arterial blood flow is reversed. In our case, the patient had characteristic advanced optic nerve cupping and pronounced visual field loss, bilateral dilated episcleral veins and an open angle with visualized blood in Schlemm’s canal, along with negative local and general findings. Thus, IDEV was one of her diagnoses.

Early IEEVP or IDEV lacks obvious symptoms and signs except for a red eye, therefore patients with this disease are easily misdiagnosed with chronic conjunctivitis, a more common abnormality in outpatient clinics. Our patient had red eyes for more than 30 years and her condition had been treated as conjunctivitis during this entire time period. When she presented to our hospital, her IOPs were over 30 mmHg with advanced glaucoma optic nerve damage, suggesting that the patient had endured episcleral vessel dilation and increased IOP for a long time, during which time the elevated pressure was not addressed. Sometimes vessel dilation cases may be caused by overuse of vaso-constrictive agents for red eyes, yet our patient had no such a history [6]. The main identifiers of IDEV are dilated and tortuous episcleral veins, rather than conjunctival hyperemia and chemosis. Spontaneous blood in Schlemm’s canal can often be found in these patients, which is a general sign of elevated episcleral venous pressure, but this is not present in all cases of IEEVP or IDEV [3].

Because no clear aetiology has yet to be identified for IEEVP or IDEV, their treatments are similar to those for primary open angle glaucoma (POAG) [7]. Suppression of aqueous humor production, medication to increase uveoscleral outflow, trabeculectomy and sclerectomy all can be used on these patients. There is a slight difference between POAG and IEEVP in treatment. Owing to episcleral venous pressure elevation, medications or methods that increase aqueous humor outflow through trabecular drainage pathway, such as pilocarpine, selective laser trabeculoplasty (SLT) and MLT are of low-efficacy. This may partially explain why the IOP of our patient’s right eye did not decrease after MLT treatment. Patients generally require a surgical procedure for refractory IOP when uncontrolled by drugs, and trabeculectomy might be a the procedure of choice. It has been reported that eyes undergoing filtering surgery are at high risk for postoperative uveal effusion [3, 8]. Therefore, taking appropriate precautions to prevent hypotony during surgery is imperative. In our case, it included maintenance of an appropriate IOP during the procedure by injection of balanced salt solution and viscoelastic agent in anterior chamber, and the application of adjustable sutures on the scleral flap to decrease the probability of excessive filtering postoperatively. Meanwhile, we used cycloplegic, oral and topical glucocorticoids to ease inflammation and to reduce the risk of choroidal effusion. Since our patient was of a younger age, anti-metabolites to reduce scarring were used before and after surgery. All of these measures might have contributed to the satisfactory result in our patient. The dilated episcleral vessels and visualization of blood in Schlemm’s canal remained unchanged in our patient after surgery, which is consistent with other reports [9]. Mark P. Breazzano reported on a young man with unilateral IEEVP, in whom dilated episcleral vessel and visualization of blood in Schlemm’s canal resolved spontaneously after anti-glaucoma medications. The author ascribed the cause of IEEVP in their case to a transient obstruction or a fistula causing temporary ocular hypertension and venous congestion [10].

## Conclusions

IEEVP or IDEV is characterized by dilated episcleral vessels and open-angle glaucoma without an underlying cause. Only by excluding other possible conditions, in particular a non-typical cavernous-sinus fistula, for which the diagnosis can be eliminated through cerebral angiography and orbital color Doppler evaluation, can the clinical diagnosis of IEEVP or IDEV be established.

This case illustrates some important issues. First, it is essential that the clinician evaluate thoroughly patients who present with episcleral vessel dilation and non-inflamed eyes. And, that the red eye characteristics are documented, and that IOPs and fundi are routinely checked, even in a fast-paced outpatient department. Also, SLT and MLT, which increase aqueous humor outflow through the trabecular meshwork, are not recommended for patients with IEEVP or IDEV. Furthermore, it is imperative that the ophthalmologist control the IOP and take precautions to prevent choroidal effusion while performing ocular filtration surgery.

## Abbreviations

BCVA: Best corrected visual acuity
IDEV: Idiopathic dilated episcleral veins
IEEVP: Idiopathic elevated episcleral venous pressure
IOP: Intraocular pressure
LE: Left eye
MLT: Micropulse diode laser trabeculoplasty
OU: Both eyes
POAG: Primary open angle glaucoma
RE: Right eye
SLT: Selective laser trabeculoplasty
UCVA: Uncorrected visual acuity
